# Supplementary material for: Exercise Metabolome: Insights for Health and Performance
Source: Metabolites. 2023 May 26;13(6):694. doi: 10.3390/metabo13060694 (PMC10305288; doi:10.3390/metabo13060694)
Supplement: Supplementary file 1 [file metabolites-13-00694-s001.zip › metabolites-2344817-supplementary.pdf]

Table S1. Metabolic changes associated with acute endurance exercise.

Author (year)	Primary Aim	Profiling Platform	Biosample	Study population	Exercise intervention/ participation	Sample collection time point	Primary findings
Tetsuyuki et al. (2019)	To evaluate changes in urine metabolites in runners in a mountain ultra-marathon	Urinalysis using Uropaper urine test strips	Urine	24 people in total (21 men, 3 women); mean age (SD) 39.5 (6.8) years	Trans Japan Alps Race 2014, a 415-km mountain ultra-marathon	5 samples; before the race, at 3 checkpoints (CP) mid-race (CP1: elevation of 2,870 m, 192.8 km from the start; CP2: 1,424 m, 236.8 km; CP3: 2,610 m, 290.7 km), and at the finish line.	Findings demonstrated increased levels of ketone bodies and fatty acids following the marathon, indicating increased lipid metabolism. Raised urobilinogen levels were also noted indicating increased hemolysis.
Blackburn et al. (2020)	To investigate changing energy metabolism during fasted exercise and rest	LC-MS	Blood serum and plasma	7 participants (5 male, 2 female) with a high level of physical activity (minimum 2.5 h intense physical activity per week); mean (SD) 26 (5) years	120-min cycling protocol; 60 min at a power corresponding to 70% of the participants VO2 max, 60-min flat course time trial at maximum exertion<br>- fasted exercise	9 samples; before exercise, then every 15 min for 2 hours of exercise	Several key ketolytic metabolites and fatty acids were observed to increase in concentration against the baseline. Findings suggest a switch from glycolytic metabolism to ketolytic metabolism. They also indicate that amino acid and energy metabolism is only occurring at a low level during glycolytic metabolism and during the recovery phase when ketolytic energy metabolism dominates.
Morville et al. (2020)	To investigate the metabolic response to endurance and resistance exercise	UPLC-MS/MS	Plasma	Healthy male subjects; mean age (SD) 24 (1) years	One hour of endurance exercise (EE) on an ergometer bike and one hour of strenuous high-volume resistance exercise (RE), with 1 week of rest between the two bouts	Eight samples: Before and immediately after 1 h of the exercise bout; six additional samples during a 3-h recovery phase	Both modes of exercise induced major changes in metabolites related to cellular energy metabolism and whole-body substrate metabolism. Induction of metabolites related to glycolysis is substantially higher with RE relative to EE. Succinate is markedly induced by EE relative to RE.
Nayor et al. (2020)	To evaluate the metabolomic response to acute exercise in middle-aged adults	LC-MS/MS	Blood	411 middle-aged participants from the Framingham Heart Study (age 53±8 years, 63% women)	Maximal cardiopulmonary exercise testing on cycle ergometer	2 samples; at rest and at peak exercise	Findings show a reduction in circulating levels of metabolites associated with insulin resistance; and increases in the levels of metabolites linked to lipolysis, nitric oxide bioavailability, and adipose browning. Distinct metabolic profiles were observed to correspond to different levels of cardiopulmonary fitness.
Varga et al. (2020)	To investigate alterations in blood lipid metabolites induced by exercise in female elite endurance athletes at risk of RED-S	LC-MS	Venous blood, blood serum	38 Scandinavian female elite athletes; mean age 26.5 years. 12 individuals had low energy availability; 26 had insufficient energy availability. 27 were diagnosed with functional hypothalamic amenorrhea; 11 had eumenorrhea (healthy menstrual cycle).	Two standardized Cycle Ergometer based fitness tests designed to obtain VO2 peak values	Five samples: at fasting state and before and after two standardized exercise tests	Associations were observed between a number of lipidomic features,cortisol and standard laboratory lipids while in a fasting state. Analysis of lipid trajectories indicates that participants with menstrual dysfunction might have decreased adaptive response to exercise interventions.

Zhao et al. (2020)	To explore the metabolic response to high intensity interval training (HIIT)	LC-MS	Urine	23 healthy young soccer players; aged under 19	Yo-Yo intermittent recovery test, consisting of repeated 2×20m shuttle runs with 10s active rest period, till exhaustion	Three samples: pre-exercise, 30 min post-exercise and after 18 hours of recovery	Metabolites associated with amino acids metabolism were significantly upregulated after exercise; the changes of steroid hormone metabolism trended to downregulate after exercise and upregulate during the recovery.
Nemkov et al. (2021)	To examine the effects of high-intensity, prolonged exercise on concentrations of metabolites and lipid markers in plasma and RBC	UHPLC–MS	Plasma, RBCs	8 well-trained, male cyclists; mean age (SD) 35 (7) years	Incremental maximal cycling exercise until exhaustion, and submaximal cycling exercise on separate days	4 samples; before and 3 min after the end of maximal and submaximal cycling test	Finding highlighted oxidative stress and activation of membrane lipid remodeling mechanisms, indicating a metabolic response in RBCs to damage resulting from increased circulation and oxygen delivery during exercise.
Osswald et al. (2021)	To investigate the metabolic effect of physical activity (PA)	SESI-HRMS	Exhaled breath	Fourteen subjects (7 female and 7 male); median age [IQR] 30 [27.0, 31.0] years	Graded cycle ergometry cardiopulmonary exercise test	Continuous real-time monitoring through gas analysis	Significant changes were observed in metabolites from several pathways, including decreases in glyoxylate and dicarboxylate, TCA cycle, and tryptophan metabolic pathways.
Schenk et al. (2021)	To explore whether PA alters tryptophan (TRP) metabolism and related inflammatory markers in Prostate Cancer Patients	HPLC-MS	Blood serum	24 prostate cancer patients; mean age (SD) 64.9 (8.4) years	30-min aerobic exercise on a bicycle ergometer at 75% of individual VO2peak	2 samples; before and directly after the exercise intervention	Interaction effects for TRP, the KYN/TRP (kynurenine/tryptophan) ratio and TGF-β were observed. Findings show acute physical exercise impacts TRP metabolism in prostate cancer patients.
Stone et al. (2021)	To investigate the effect of different intensities of exercise on levels of amino acids (AA) and related metabolites	N/A	Blood, sweat	10 subjects	Maximally ramped exercise test and three 30-min submaximal (45/60/75% VO2max) exercise bouts	2 blood samples; before/after the exercise bouts. Sweat was collected from the forearm throughout.	Plasma concentrations were not significantly different between exercise bouts performed at 45 and 60%. Exercise at 75% tended to reduce concentrations of sweat amino acids. Findings suggest increasing exercise intensity increases AA metabolism as demonstrated by reduced plasma AA concentrations and increased excretion through sweat glands.
Chen et al. (2022)	To understand the metabolic changes of athletes in response to exercise	NMR	Urine	14 male athletes in middle- and long-distance running groups; mean age (SD) 17.33 (1.58) years	Endurance and speed endurance training: 8000 m running, (500 m fast running +100 m jogging) x16 times	2 samples: before and 30 min after training	Significant metabolic differences were observed in the samples before and after training, with the greatest differences arising from increased lactic acid, glycine, and n-trimethylamine oxide concentrations, and a decreased creatinine concentration.
Li et al. (2022)	To analyze the metabolic changes in response to acute endurance exercise	LC-MS/MS	Cerebrospinal fluid (CSF), plasma	19 young active adults (13 males and 6 females)	90-min monitored outdoor run	2 samples: before and 60 min after running	Metabolic pathways of amino acid, nucleotide, and fatty acid metabolism were observed to be activated. More than 80% of the changes in the CSF were linked to mitochondrial and metabolic changes caused by ATP signaling.
Nelson et al. (2022)	To evaluate the metabolic response to acute aerobic exercise	LC-MS	Serum	25 overweight/obese trained and normal weight trained runners	90-minute treadmill run at 60% VO2max	2 samples: immediately before and after exercise	FAHFAs decreased with acute aerobic exercise in normal weight trained runners but not with those that were overweight/obese - indicating

							FAHFAs could influence the inflammatory response, fuel utilization, and insulin resistance.
Pellegrino et al. (2022)	To evaluate the metabolic response to acute bouts of aerobic or anaerobic exercise in males and females	MS/MS	Serum	40 healthy, active participants; mean age (SD) 24 (3.1) years	45 min of aerobic cycling or resistance exercise	3 samples: rest, immediately after and 1-hour post-exercise	The two exercise metabolomes were fairly similar; both significantly altered from rest following exercise and returned toward baseline following the recovery period. Faster recovery observed following aerobic cycling can be explained by elevated lipid metabolism.
Pinto et al. (2022)	To evaluate differences between the redox, hormonal, metabolic, and lipid profiles of female and male basketball players during the seasonal training period, versus their sedentary controls	GC–MS, LC–MS/MS	Capillary blood and saliva	10 female professional basketball players, 10 male professional basketball players, and the respective sedentary controls (10 females and 10 males)	30 min of low-moderate running, followed by interval training runs	One sample: 48 h after the last competition and 24 h after the training session	Antioxidant potential enhancement, lactate increase, and activation of urea cycle and arachidonic pathways in response to inflammation was evident in both sexes. Lipid and amino acid utilization differed between sexes.
Zagatto et al. (2022)	To evaluate the metabolic response in muscle tissue to exhaustive high intensity cycling exercise	H-NMR	Muscle	7 healthy, physically active men; mean age (SD) 21 (2) years	Graded cycling exercise test and an exhaustive supramaximal effort at 115% of maximal aerobic power	2 samples: at rest and immediately after exhaustion	High-intensity exercise was associated with glycolytic and phosphagen energetic pathways, in addition to pathways indicative of amino acid and fatty acid metabolism.

Table S2. Metabolic changes associated with subacute endurance exercise.

Author (year)	Primary Aim	Profiling Platform	Biosample	Study population	Exercise intervention/ participation	Sample collection time point	Primary findings
Chlíbková et al. (2018)	To examine the effect of running seven marathons in 7 consecutive days on fluid metabolism	N/A	Plasma, Urine	6 women and 20 men; age (SD) 42.6 (6.2)	Seven marathons in seven consecutive days	4 samples; before day 1 and after day 1, 4, and 7	Plasma sodium, plasma potassium and urine sodium remained stable at pre-race (baseline) levels; urine urea increased, which may be related to increased protein catabolism.
Kistner et al. (2019)	To investigate the effect of HIIT on the resting urinary metabolome of young active men	NMR, LC-MS	Urine	20 healthy, regularly active men, aged between 20 and 50 years	10-day HIIT consisting of progressive exercise test on bicycle ergometer until exhaustion	2 samples; before and after exercise intervention	No overall change in resting urinary metabolome, except a significant difference with decreasing means in urinary hypoxanthine concentration, which may indicate a training-induced adaptation in purine nucleotide metabolism.
Joisten et al. (2020)	To examine effect of exercise on plasma neurofilament light chain (pNfl) and kynurenine (KYN) pathway of tryptophan degradation metabolites in persons with multiple sclerosis	HPLC-MS/ MS	Blood plasma	69 men and women with multiple sclerosis (Expanded Disability Status Scale score 3.0–6.0); mean age (SD) 50.28 (10.12) years	3-week training intervention; participants randomly assigned to a high-intensity interval training (HIIT) or an moderate continuous training (MCT) group	4 samples; before, after, and 3 hours after the first training session as well as after the 3-week training intervention.	Acute exercise reduced pNfL and increased the KYN pathway flux toward the neuroprotective kynurenic acid. Effects were consistently greater with HIIT than with MCT. After the 3-week training intervention, the KYN pathway was activated in HIIT compared with MCT.
Deutsch et al. (2022)	To compare urine metabolomes of physically active and inactive healthy young males in response to physical exercise	H-NMR	Urine	20 participants; 10 in trained group - mean age (SD) 23 (2) years; 10 in untrained group mean age (SD) 25 (3) years	10-day training protocol consisting of 1 h of 50% maximal pedaling power output per day	2 samples: before and after exercise intervention	Key metabolic differences in the trained and untrained group arose from concentrations of cholate, tartrate, cadaverine, lysine and N6-acetyllisine. Following the exercise intervention, the urine metabolome of the untrained group was effectively modified to one identical to the trained group.
Savikj et al. (2022)	To evaluate metabolic effects of exercise in men with type 2 diabetes.	UPLC-MS	Blood, subcutaneous adipose tissue	15 men with type 2 diabetes (age 45-68 years and body mass index 23-33 kg/m2)	2 weeks of HIIT training (three sessions/week) either in the morning (08:00, n = 5) or afternoon (16:45, n = 3), a 2-week wash-out period, followed by two more weeks of HIT at the opposing time	3 samples: at the start of the study and 3 days after the last round of each HIIT period	Both morning and afternoon sessions of HIIT showed increases in plasma diacylglycerols, skeletal muscle acyl-carnitines, and subcutaneous adipose tissue sphingomyelins and lysophospholipids , as well as changes in adipose tissue lipid composition.

Table S3. Metabolic changes associated with chronic endurance exercise.

Author (year)	Primary Aim	Profiling Platform	Biosample	Study population	Exercise intervention/ participation	Sample collection time point	Primary findings
Brennan et al. (2018)	To investigate the changes to adipose tissue metabolic profiles following aerobic exercise	LC-MS/MS	Plasma	103 middle-aged abdominally obese men and women; mean (SD) 52.4 (8.0) years	24 weeks in one of four aerobic exercise interventions: control, low amount low intensity, high amount low intensity, and high amount high intensity	2 samples; prior to and 48 hours after the exercise intervention	Aerobic exercise was found to induce alterations in the levels of intermediates of the TCA cycle and amino acid metabolism. Findings also pointed to potential biomarkers of adipose tissue reduction which may mediate cardiometabolic improvements.
Brennan et al. (2018)	To investigate the metabolic response to chronic exercise	LC-MS/MS	Plasma	216 middle-age abdominally obese men and women; mean (SD) 52.4 (8.0) years	24 weeks in one of four aerobic exercise interventions: control, low amount low intensity, high amount low intensity, and high amount high intensity	3 samples; baseline, and after the last exercise session at 16 and 24 weeks	Significant changes were observed in the levels of metabolites involved in tryptophan metabolism, energy metabolism, pyruvate metabolism, fatty acid synthesis, and purine degradation in the exercise groups, predictive of cardiometabolic improvements.
Ahmeti (2020)	To compare the metabolic profiles induced by endurance versus resistance training	LC-MS/MS	Blood	57 women (age: 23 ± 3 years); divided into endurance training group (n=20) and resistance training group (n=19)	8 weeks of either endurance (treadmill walking/running) or resistance training (circuit weight training); 3 training sessions a week	2 samples: before and after 8-week intervention	Both endurance and resistance training showed positive effects on lipid profiles, including a decreased concentration of triglycerides, increased HDL, as well as reduced plasma glucose. indicating improvements in cardiovascular risk factors.. No differential effects were noted.
Chacaroun et al. (2020)	To compare the effect of hypoxic and normoxic exercise training on cardiometabolic parameters in obese individuals	N/A	Blood plasma	23 subjects (11 men and 1 woman); mean age (SD) 52 (12) years	8-week hypoxic exercise training program with 3 sessions per week; constant-load cycling at 75% of maximal heart rate	2 samples; before and after the training program	No change in vascular function and metabolic status was observed after training. Hypoxic exercise training only increased nitrite and reduced superoxide dismutase concentrations.
Serra et al. (2020)	To evaluate plasma metabolomic profiles in chronic stroke survivors	HPLC-MS	Plasma	25 chronic stroke survivors; mean age (SD) 62 (1)	Randomly assigned to 6 months of treadmill exercise (N = 17) or	2 samples; before and after intervention	Significant enrichment in 4 pathways following treadmill exercise, 3 of which (heparan-, chondroitin-, keratan-sulfate degradation) involved connective

	following aerobic exercise training				whole-body stretching as a nonaerobic control (N = 8)		tissue metabolism and the fourth involved lipid signaling (linoleate metabolism)
König et al. (2021)	To analyze the metabolome of long-distance runners, and evaluate the correlation of the metabolome with exercise performance	MS	Urine	24 participants (4 females, 20 males)	TransEuropeFootRace ultramarathon: participants ran 4487 km over 64 days (chronic):	Between 1 to 5 samples taken for each participant, on different days during the race	Results indicated metabolic profiles to be distinct amongst race finishers and non-finishers, with differences in energy metabolism, reparative- and/or detoxifying and (anti-) inflammatory processes. Changes of proteolysis, catabolism, and high oxidative stress were observed.
Pintus et al. (2021)	To study the metabolic response to PA in athletes	N/A	Blood, saliva, urine	21 professional soccer players*	Preseason soccer training	3 samples; at 3 different time points during the preseason preparation period before the beginning of Serie A Championship in Italy.	Urine profile changed during the observational period. Significant variation observed for trimethylamine-N-oxide, dimethylamine, hippuric acid, hypoxanthine, guanidoacetic acid, 3-hydroxybutyric acid, citric acid and creatine; these could be related to diet, training and microbiota.
Schranner et al. (2021)	To study the effect of acute exercise on the metabolome of highly glycolytic, oxidative, and anabolic athletes (sprinters, endurance athletes, and natural bodybuilders)	LC-MS/MS, FIA-MS/MS	Serum	15 competitive male athletes (6 endurance athletes, 5 sprinters, and 4 natural bodybuilders) and 4 untrained control subjects	Maximum graded bicycle test to exhaustion for athletes of different sport categories (endurance, natural bodybuilders, sprinters)	2 samples; at fasted rest and 5 minutes after intervention	Endurance athletes and natural bodybuilders were associated with unique blood metabolite concentrations and ratios. Natural bodybuilders had 1.5 - 1.8 fold higher concentrations of phosphatidylcholines and lower levels of branched chain amino acids. Endurance athletes had 1.4-fold higher levels of carnitine-palmitoyl-transferase I and 1.4-fold lower levels of various alkyl-acyl-phosphatidylcholines.
Cruz et al. (2022)	To compare the metabolome of U22 soccer players according to their competitive level	LC-MS	Capillary blood	36 male U22 soccer players (non-elite = 20 athletes, elite = 16 athletes)	U22 football national league (players in second week of preseason training)	4 samples following a 12-h overnight fast	Findings show significant metabolic differences between players of elite and non-elite teams. Metabolites related to aerobic power, greater efficiency in the recovery process, and immunity were in higher abundance in the elite group.
Hintikka et al. (2022)	To analyze the serum metabolome of elite cross-country skiers	NMR	Serum	27 athletes from the national Nordic Ski Team - mean age	Elite cross-country skiing on the national Nordic Ski Team, following 11 month	One sample: the day before the competition	Athletes had a healthier serum lipid profile than the controls, a higher concentration of pyruvate, and lower concentrations of ketone bodies.

				(SD) 27.1 (5.1) years - and 27 controls	training and competitive season		
Tarkhan et al. (2022)	To compare metabolic profiles of female endurance athletes to non-athletes	UHPLC-MS	Serum	51 elite female endurance athletes and 197 non-athletic females	Elite endurance sports	One sample	Elite female endurance athletes displayed a distinct steroid hormone profile to that of non-athletes, with significant differences in their androgen and corticosteroid levels.
Vike et al. (2022)	To investigate the metabolic response to contact sports participation	UPLC–MS/MS	Serum	23 male collegiate football athletes; mean age (SD) 21 (1) years	Collegiate football athletic season	2 samples: before the athletic season (Pre) and after the last game (Post)	Findings show significant alterations in metabolites involved in five pathways - xanthine, fatty acid (acyl choline), medium chain fatty acid, primary bile acid, and glycolysis, gluconeogenesis, and pyruvate metabolism – post-season.

Table S4. Metabolic changes associated with acute resistance exercise.

Author (year)	Primary Aim	Profiling Platform	Biosample	Study population	Exercise intervention/ participation	Sample collection time point	Primary findings
Pechlivanis et al. (2015)	To monitor the response of the human urinary metabolome to short maximal exercise	RP-UPLC-MS, H NMR	Urine	17 young, physically active, healthy male volunteers; age (SD) 19 (1) years	2 identical sprint sessions on separate days, consisting of three 80 m maximal runs	4 samples; before exercise, 1, 1.5, and 2 h postexercise	Findings show sustained increase in the levels of the purine degradation products, hypoxanthine and inosine, suggestive of a major stress on ATP metabolism. Overall, results show even 1.5 minute of maximal exercise elicited major perturbations in human metabolism, several of which persisted for at least 2 h.
Feriche et al. (2019)	To study the effects of exercise at moderate altitude on metabolites linked to muscle growth	Photometric procedure (Dr. Lange, LP 20 plus, 177 Berlin, Germany)	Blood	13 male volunteers, who were experienced lifters and lived at low altitude: age (SD) 22.31 (2.59) years	2 standard hypertrophic resistance training sessions, one at moderate terrestrial altitude and the other under normoxic conditions	4 samples; before and after the exercise sessions at hypoxic and normoxic conditions	Findings do not support an accentuated effect of exercise at moderate altitude on metabolic factors linked to muscle growth during resistance training.
Fiorenza et al. (2019)	To investigate changes in muscle metabolism in	Fluorometric analyses	Muscle, blod	11 endurance-trained men; mean age (SD) 31.6 (2.6)years	High-intensity intermittent cycle exercise	8 samples; immediately after the first sprint, at 33% and 66% of exercise task completed, 10 s	Long-sprint exercise showed more extensive intramuscular accumulation of lactate/H+ and lower rates of

	trained individuals during multiple long and short duration sprints.				protocols: multiple short- or long-duration sprints.	before and immediately after the last sprint, and after exercise at 3 min and 5 min into recovery	glycolysis than short sprint, indicating long sprints induce greater peripheral fatigue and impairments in performance .
Morville et al. (2020)	To investigate the metabolic response to endurance and resistance exercise	UPLC-MS/MS	Plasma	Healthy male subjects; mean age (SD)<br>24 (1) years	One hour of endurance exercise (EE) on an ergometer bike and one hour of strenuous high-volume resistance exercise (RE), with 1 week of rest between the two bouts	Eight samples: Before and immediately after 1 h of the exercise bout; six additional samples during a 3-h recovery phase	Major changes observed in metabolites related to cellular energy metabolism and whole-body substrate metabolism. Induction of metabolites related to glycolysis is substantially higher with RE relative to EE. Succinate is markedly induced by EE relative to RE.
Mieszkowski et al. (2021)	To compare exercise-induced changes in vitamin D metabolites and bone metabolism in athletes and non-athletes	Serum CTX, PINP, and vitamin D assays	Serum	Sixteen elite male artistic gymnasts; mean age (SD) 21.4 (0.8) years.<br><br>16 physically active men in control group; mean age (SD) 20.9 (1.2)	Lower and upper body 30-s Wingate anaerobic tests	6 samples; before, and 5 and 30 min after both the Lower Body and Upper Body Wingate tests	Vitamin D metabolites were found to affect the anaerobic performance and bone metabolic markers at rest and after exercise. Moreover, adaptation to physical activity was found to regulate effect of anaerobic exercise on bone metabolismmarkers.
Dünnwald et al. (2022)	To investigate the metabolic response to concentric-eccentric exercise under hypoxia	H-NMR	Blood plasma	11 male trained alpine ski athletes; mean age (SD) 30.4 (6.0) years	Concentric-eccentric leg exercises on flywheel device (5 sets, each lasting 90 secs)	3 samples: before, 20 min after exercise, and on day 8 following intervention	Findings show significant differences in metabolites involved in carbohydrate, TCA, amino acid, and purine metabolism immediately post-exercise. Some of these changes were shown to persist on day 8 following the exercise.
Pellegrino et al. (2022)	To evaluate the metabolic response to acute bouts of aerobic or anaerobic exercise in males and females	MS/MS	Serum	40 healthy, active participants; mean age (SD) 24 (3.1) years	45 min of aerobic cycling or resistance exercise	3 samples: rest, immediately after and 1-hour post-exercise	The two exercise metabolomes were fairly similar; both significantly altered from rest following exercise and returned toward baseline following the recovery period. Faster recovery observed following aerobic cycling can be explained by elevated lipid metabolism.

Table S5. Metabolic changes associated with chronic resistance exercise.

Author (year)	Primary Aim	Profiling Platform	Biosample	Study population	Exercise intervention/ participation	Sample collection time point	Primary findings
Ahmeti (2020)	To compare the metabolic profiles induced by endurance versus resistance training	LC-MS/MS	Blood	57 women (age: 23 ± 3 years); dividded into endurance training group (n=20) and resistance training group (n=19)	8 weeks of either endurance (treadmill walking/running) or resistance training (circuit weight training); 3 training sessions a week	2 samples: before and after 8-week intervention	Both endurance and resistance training showed positive effects on lipid profiles, including a decreased concentration of triglycerides, increased HDL, as well as reduced plasma glucose, incdicating improvements in metabolites linked to cardiovascular risk factors . No differential effects were noted.
Schranner et al. (2021)	To study the effect of acute exercise on the metabolome of highly glycolytic, oxidative, and anabolic athletes (sprinters, endurance athletes, and natural bodybuilders)	LC-MS/MS, FIA-MS/MS	Serum	15 competitive male athletes (6 endurance athletes, 5 sprinters, and 4 natural bodybuilders) and 4 untrained control subjects	Maximum graded bicycle test to exhaustion for athletes of different sport categories (endurance, natural bodybuilders, sprinters)	2 samples; at fasted rest and 5 minutes after intervention	Endurance athletes and natural bodybuilders were associated with unique blood metabolite concentrations and ratios. Natural bodybuilders had 1.5 - 1.8 fold higher concentrations of phosphatidylcholines and lower levels of branched chain amino acids. Endurance athletes had 1.4-fold higher levels of carnitine-palmitoyl-transferase I and 1.4-fold lower levels of various alkyl-acyl-phosphatidylcholines.
Shen et al. (2021)	To explore metabolic effects of Tai-Chi in subjects with knee osteoarthritis	UHPLC-MS	Plasma	12 postmenopausal women with knee osteoarthritis (>50 years of age)	8-week Tai-Chi group intervention	2 samples: before and after 8 weeks of intervention	Findings show significant metabolic differences suggesting increased fatty acid catabolism, protein turnover and changes in lipid redistribution follwing Tai-Chi intervention
Gehlert et al. (2022)	To determine metabolic effects of acute resistance exercise (RE) in human skeletal muscle	LC-MS	Muscle	6 healthy male untrained volunteers	13 RE training sessions over 5 weeks	3 samples: at rest, 45 min after a first bout of RE (untrained state), and 45 min after the last bout of a five-week-long resistance training program (trained state)	Acute RE induced significant changes in the levels of metabolites involved in amino acid, purine, primary bile acid, and nicotinate and nicotinamide metabolism,. Chronic RE was found to alter the levels of metabolites involved in amino acid, nicotinate and nicotinamide, and lipid metabolism.

Table S6. Metabolic changes associated with combined endurance-resistance exercise.

Author (year)	Primary Aim	Profiling Platform	Biosample	Study population	Exercise intervention/ participation	Sample collection time point	Primary findings
Wang et al. (2021)	To investigate changes in urine metabolism in female water polo players before and after high-intensity training	LC-MS	Urine	12 young female water polo players; mean age (SD) 16.46 (1.56) years	4-week formal training started after 1 week of acclimatization	Three samples: Before formal training, early morning after 4 weeks of training, and immediately after 4 weeks of training matches	Alterations were observed in the levels of metabolites involved in three metabolic pathways: niacin and niacinamide, purine, and histidine metabolism.
Zhou et al. (2021)	To examine the metabolic response to exercise in female athletes	GC-MS	Urine, Serum	12 female adolescent volleyball athletes	2-week strength-endurance training	2 samples; before and after exercise intervention	Findings show significant changes in metabolites primarily involved in energy metabolism, lipid metabolism and amino acids metabolism. Results also indicate disordered hormone adjustment and exercise-induced oxidative stress.
Loureiro et al. (2022)	To determine the metabolic impact of precompetitive training in modern pentathletes	NMR	Blood serum	6 members of a Brazilian modern pentathlon team	3-week training period before pentathlon competition	6 samples: at the beginning (Pre1, Pre2, and Pre3) and end (Post1, Post2, and Post3) of each week	Significant metabolic changes were observed, depending on the intensity of the training performed each week. Of the main metabolic changes – alterations in the levels of lactate, creatine kinase, peroxides, and sarcosine - sarcosine showed the greatest pretraining and post training differences.

Table S7. Metabolic changes associated with combined chronic endurance-resistance exercise.

Author (year)	Primary Aim	Profiling Platform	Biosample	Study population	Exercise intervention/ participation	Sample collection time point	Primary findings
Baldassari et al. (2018)	To investigate the effect of exercise intervention on the NO (nitric oxide) pathway in patients with heart failure with preserved ejection fraction (HFpEF)	LC-MS/MS	Plasma	62 patients with heart failure with preserved ejection fraction; mean age (SD) 65 (6) years	3 months of endurance training (bicycle Ergometer) and resistance training (bench press, leg press, leg curl, rowing, triceps dip)	2 samples; before and after intervention	Exercise intervention did not influence NO pathway parameters in HFpEF patients, but the metabolite L-hArg was related to change in peak V̇O2.
Duft et al. (2018)	To investigate changes in the metabolism of obese men induced by PA	NMR	Blood	22 obese middle-aged men; mean age (SD) 48.2 (6.1) years	Resistance and aerobic training, 3 times a week for 24 weeks	2 samples; before and after intervention	24 weeks of training program were effective for functional improvements and metabolic changes in obese middle-aged men. Tyrosine, 2-oxoisocaproate, histidine, pyruvate classified as best discriminators; some metabolites correlated with strength, VO2 peak, fat and lean body mass, waist circumference, and insulin.

Duft et al. (2020)	To investigate metabolic response to combined training (CT) in overweight and obese adolescents	NMR	Serum	37 overweight and obese adolescents of both sexes; mean age (SD) 14.6 (1.05) years	60 min of combined endurance (30 min walking/running on treadmill) and resistance training, 3 times a week for 12 weeks	2 samples; before and after intervention	Findings demonstrate the positive effects of CT program on metabolomic profile and glucose metabolism in overweight and obese adolescents. Significant changes were observed in the levels of the2-oxoisocaproate, 3-hydroxyisobutyrate, glucose, glutamine and pyruvate.
Koay et al. (2021)	Significant reduction in the fuel substrates, fatty acids and ketone bodies. Highly significant changes of amino acid, lipid, and nucleotide metabolism.	LC-MS	Plasma	52 newly enlisted male recruits (22 ± 4 years)	80-day moderate-intensity mixed aerobic and strength exercise programme chronic mixed	2 samples; at rest and at peak exercise	Findings show a reduction in the concentrations of metabolites associated with insulin resistance; and increases in the concentrations of metabolites linked to lipolysis, nitric oxide bioavailability, and adipose browning. Distinct metabolic profiles were observed to correspond to different levels of cardiopulmonary fitness.
Lee et al. (2021)	To investigate the effect of long-term exercise on branched-chain amino acid (BCAA) metabolism in sedentary dysglycemic and normoglycemic men	GC-MS/MS, HPLC-MS/MS	Plasma	26 sedentary men aged 40 to 65 years of Scandinavian Origin - 13 of whom were dysglycemic, and 13 who were normoglycemic	12 weeks of supervised intensive exercise intervention, including two whole-body strength training sessions (resistance) and two bicycle interval sessions (endurance) of 1h each	2 samples; before and after 12-week intervention*	Plasma concentrations of BCAAs and related metabolites remain largely unaltered after 12 weeks of PA. Long-term exercise may regulate BCAA metabolism in muscle and adipose tissue through altered expression of the genes encoding BCKDH kinase and phosphatase, and the SLC25A44 mitochondrial BCAA transporter.
Mendham et al. (2021)	To explore relationship of metabolic signature in response to aerobic and resistance exercise with mitochondrial respiration and insulin sensitivity in women with obesity	GC-TOF/MS, LC-TOF/MS	Serum, muscle	45 obese sedentary black South African women; 20–35 years of age	12 weeks of combined aerobic and resistance exercise training	2 samples: before and after intervention	Findings showed significant changes to the skeletal muscle lipid profile, marked by increased diacylglycerol and ceramide levels. Metabolic changes were associated with increases in mitochondrial respiration.
Militello et al. (2021)	To determine the metabolic profile and redox profile of elite female basketball players during the	GC-MS	Plasma	10 female professional basketball players and 10 controls; mean (SD) age 26 (4.13) years	Athletes performed five 2 h-lasting training sessions per week; controls were sedentary	5 samples per week throughout training season; collected morning before training session	Trained women showed greater antioxidant capacity and a reduction in oxidative species than sedentary women

	seasonal training period						
Cai et al. (2022)	To compare the metabolome of elite and sub-elite swimmers	NMR	Blood	103 elite and 84 sub-elite level Chinese professional swimmers	Chinese professional swimming (swimmers in post competition recovery period)	One sample in the morning after overnight fasting	Findings indicate increased amino acid, lipid, and carbohydrate metabolism.
Sardeli et al. (2022)	To evalute the metabolic response to combined aerobic and resistance exercise training in older women with metabolic syndrome (MetS)	H-NMR	Serum	12 older women with MetS and 13 in the control group	16-week combined aerobic and resistance exercise training; consisting of walking/running at 63% of VO2max, and resistance training	5 samples: baseline (0W), 4 (4W), 8 (8W), 12 (12W) and 16 weeks (16W)	Findings showed increased levels of the substrate to the tricarboxylic acid cycle, and alterations in the production of ketone bodies, which may explain the improved fatty acid metabolism associated with exercise.
